# Crosstalk between colorectal CSCs and immune cells in tumorigenesis, and strategies for targeting colorectal CSCs

**DOI:** 10.1186/s40164-024-00474-x

**Published:** 2024-01-22

**Authors:** Qi Zhao, Hong Zong, Pingping Zhu, Chang Su, Wenxue Tang, Zhenzhen Chen, Shuiling Jin

**Affiliations:** 1 Department of Oncology, The First Affiliated Hospital of Zhengzhou University, Zhengzhou, 450052 China; 2 School of Life Sciences, Zhengzhou University, Zhengzhou, 450001 China; 3 The Research and Application Center of Precision Medicine, The Second Affiliated Hospital of Zhengzhou University, No. 2 Jing‑ba Road, Zhengzhou, 450014 China

**Keywords:** Colorectal cancer stem cells, Immune Cells, Tumor immune microenvironment, Targeting cancer stem cells, Immunotherapy

## Abstract

Cancer immunotherapy has emerged as a promising strategy in the treatment of colorectal cancer, and relapse after tumor immunotherapy has attracted increasing attention. Cancer stem cells (CSCs), a small subset of tumor cells with self-renewal and differentiation capacities, are resistant to traditional therapies such as radiotherapy and chemotherapy. Recently, CSCs have been proven to be the cells driving tumor relapse after immunotherapy. However, the mutual interactions between CSCs and cancer niche immune cells are largely uncharacterized. In this review, we focus on colorectal CSCs, CSC-immune cell interactions and CSC-based immunotherapy. Colorectal CSCs are characterized by robust expression of surface markers such as CD44, CD133 and Lgr5; hyperactivation of stemness-related signaling pathways, such as the Wnt/β-catenin, Hippo/Yap1, Jak/Stat and Notch pathways; and disordered epigenetic modifications, including DNA methylation, histone modification, chromatin remodeling, and noncoding RNA action. Moreover, colorectal CSCs express abnormal levels of immune-related genes such as MHC and immune checkpoint molecules and mutually interact with cancer niche cells in multiple tumorigenesis-related processes, including tumor initiation, maintenance, metastasis and drug resistance. To date, many therapies targeting CSCs have been evaluated, including monoclonal antibodies, antibody‒drug conjugates, bispecific antibodies, tumor vaccines adoptive cell therapy, and small molecule inhibitors. With the development of CSC-/niche-targeting technology, as well as the integration of multidisciplinary studies, novel therapies that eliminate CSCs and reverse their immunosuppressive microenvironment are expected to be developed for the treatment of solid tumors, including colorectal cancer.

## Introduction

Colorectal cancer (CRC) is the third leading cause of cancer-induced death, with 1.85 million cases and 850 000 deaths every year [1].Previously, The incidence of CRC was higher in developed countries than that in developing countries. However, with lifestyle changes, such as irregular sleeping,Westernized diets, and improvements in living standards, the incidence and mortality rates of CRC patients are increasing in many developing countries [2]. Like many other types of tumors, CRC is characterized by heterogeneity, including intertumoral heterogeneity and intratumoral heterogeneity. Different patients, and even different cells within the same tumor, respond differently to the same treatment, greatly increasing the difficulty of tumor therapy [3].Among different types of tumor cells, cancer stem cells (CSCs) have attracted increasing attention.

Cancer stem cells constitute small subset of tumorigenic cells in tumors with the ability to self-renew and differentiate into heterogeneous tumor cells [4]. CSCs play important roles in tumor initiation, maintenance, metastasis and drug resistance and hence are closely related to patient survival. CSCs are generally in a dormant state and express various drug pumps at high levels and thus are insensitive to chemotherapeutics, leading to drug resistance and disease relapse [5]. Moreover, accumulating evidence demonstrates that CSCs are resistant to immune surveillance and immunotherapy [6]. Immune escape of CSCs is a prerequisite for tumor initiation, and CSCs resistant immunotherapy for various reasons, including low MHC expression and high PD-L1 expression [7, 8]. The immune cells play a crucial role in the CSC niche, and CSCs are key modulators of immune niche remodeling [9].

Herein, we review recent progress in colorectal CSCs and immune niches, including CSC surface markers, signaling pathways, epigenetic modifications, especially CSC-immune cell crosstalk in colorectal tumors and CSC-targeted strategies.

## Colorectal CSCs

### Surface markers

To some extent, the study of CSCs originates from the identification of their surface markers. CSC surface markers are particularly important for the identification, isolation, characteristic analysis and eradication of CSCs. Several CSC surface markers have been identified in patients with CRC, the identification of CD44, CD133, Lgr5 and DCLK1 as CRC biomarkers has been widely accepted.

#### CD44

CD44 is a multifunctional transmembrane glycoprotein encoded by a gene on human chromosome 11. The CD44 gene contains 19 exons. The first 5 and last 5 exons stably encode CD44 standard isoforms (CD44s). The middle 9 exons are alternatively spliced and assembled into 10 exons to form CD44 variant isoforms (CD44v) [10, 11]. CD44 is a receptor for hyaluronic acid (HA) and multiple cytokines that mediates cell–cell and cell–matrix adhesion. It has been identified as a surface marker for a variety of CSCs, including CRC CSCs [12]. CD44 functions as a positive regulator of the Wnt/β-catenin signaling pathway, serving as a modulator in the location and activation of the Wnt receptor LRP6 [13]. Knocking down CD44 can inhibit the proliferation and migration of CRC cells and can promote cell apoptosis [14]. CD44^+^ cells harbor an enhanced tumor initiation capacity. A single CD44^+^ cell can proliferate into stem-like tumor spheres in vitro, and tumor spheres xenografted into nude mice can develop into tumors [15]. The CD44-Src-integrin axis promotes the formation and survival of CRC cells in vitro. In addition, translocated nuclear CD44/acetylated STAT3 reprograms CRC cells to express stemness characteristics by regulating the expression of c-myc [16]. CD44 is associated with prognosis and metastasis in CRC patients [17]. CRC CSCs express CD44v6, a variant isoform of CD44, which acts as a coreceptor for MET to activate the EMT program and promote colorectal cancer metastasis and is associated with poor prognosis in CRC patients [18, 19]. In addition, PrPc^+^CD44^+^ CSCs (a subset of CD44^+^ CSCs) promote EMT through the ERK2 pathway, making tumors highly metastatic [20]. Moreover, CD44 is a functional target for colorectal CSCs elimination. CD44 silencing prevents tumorigenesis and clonal formation [15].

#### CD133

CD133, a five-time transmembrane glycoprotein encoded by the *prominin 1* gene on human chromosome 4, was originally found in human hematopoietic stem cells and progenitor cells. It has been recognized as a marker for CSC in various solid tumors, such as liver, colorectal, prostate, and pancreatic tumors [21]. Ricci-Vitiani demonstrated that a small subset (approximately 2.5%) of tumor cells with high CD133 expression were CSCs. Compared with CD133^−^ cells, CD133^+^ cells isolated from primary CRC samples showed enhanced long-term tumorigenic potential, and their CSC characteristics were diminished via serum-induced differentiation [22]. On the contrary, Shmelkov SV et al. found that CD133 expression was not limited to the CSCs of CRC, but also present in normal colonic epithelial cells. Both CD133^+^ and CD133^−^ cells isolated from metastatic CRC can initiate tumorigenesis, and the tumors produced by the CD133^−^ cell subsets are even more aggressive [23]. Through exploiting CD133-Cre-lacZ model mice, Zhu et al. revealed that CD133 was an optimal marker of CSCs in the small intestine but could not be used as a colorectal CSC marker in these mice. The widespread CD133 expression in the colon, including in stem cells and multiple differentiated cells such as goblet cells, has prevented CD133 from being used as a colorectal CSC marker in model mice [24]. Notably, although the suitability of CD133^+^ CSCs as a marker in the mouse colon remains debated, the use of CD133 as a CSC marker in human CRC has been widely accepted. In vitro, CD133^+^ human CRC cells can form tumor spheres, and xenotransplantation of these tumor spheres into immunodeficient mice results in tumor development. Additionally, CD133^+^ cells can secrete IL-4 to resist cell death and exhibit resistance to chemotherapy [25]. CD133 expression is also associated with poor survival in CRC patients [26]. Compared with CD133^−^ CRC cells, CD133^+^ CRC cells have higher AKT and MAPK pathway activity. [27, 28]. Targeting CD133 can reverse the chemotherapy resistance of CRC cells through the AKT/NF-κB/MDR1 pathway [29].

#### Lgr5

Lgr5 is a seven transmembrane domain receptor in the G protein-coupled receptor rhodopsin family. First recognized as a marker of intestinal stem cells (ISCs) in 2007, it is also a target gene of the classical stem signaling pathway Wnt/β-catenin [30, 31]. The identity of CD44 and CD133 as colorectal CSCs remains doubtful. Some studies suggested that the expression of CD44 or CD133 may be insufficient to determine the identity of colorectal CSCs [23, 32]. Since its discovery, scientists have found that Lgr5^+^ cells in aggressive lesions of intestinal adenomas harbor CSC signatures and potentials, and are associated with tumor expansion and stemness [33]. In mouse models, conditioned knockout of APC in Lgr5^−^ cells inhibited the growth of intestinal adenomas, while conditional knockout of APC in Lgr5^+^ cells drove intestinal adenomas, proving that Lgr5^+^ cells are CSCs for colorectal tumors [34]. Clonal fate tracing and retracing analyses revealed that Lgr5^+^ cells residing in the base of an adenoma segment can undergo self-renewal and differentiation into several cell types, providing more definitive evidence that Lgr5 is a marker of adenoma CSCs [35]. Single Lgr5^+^ cell from intestinal crypts can grow into organoids with villus/crypt structures in three-dimensional cultures in vitro [36, 37]. Organoid technology based on this has played an important role in stem cell function research, disease modeling and other fields. Orthotopic transplantation of tumor organoids constructed from Lgr5^+^ CSCs into mice drove tumorigenesis, and targeted elimination of Lgr5^+^ cells inhibited tumorigenesis [38]. However, the depletion of Lgr5^+^ cells does not block the re-initiation and regression, which is related to the plasticity of CSCs. In addition, Lgr5^+^ cells have been closely associated with distant metastasis of CRC, especially in the formation and maintenance of liver metastasis [39]. Similar to Lgr5, Olfm4 and Ascl2 are also studied as intestinal stem cell markers. Since Lgr5 mRNA and protein are expressed at low levels in cells, Olfm4, as a characteristic gene of Lgr5^+^ stem cells, is often used as a substitute for Lgr5 to mark intestinal stem cells [40]. Some studies have shown that Olfm4 may not be used as an accurate marker of intestinal stem cells. Olfm4 is not only expressed in cells at the base of the crypts, but in almost all cells in the crypts [41]. However, Olfm4 has been confirmed to play an important role in CRC. In *APC* mutant mice, the deletion of *Olfm4* leads to colon adenocarcinoma [42]. The transcription factor Ascl2 is a target gene of Wnt. In CRC cases, there is a clear correlation between the expression of Lgr5 and Ascl2 [43]. Ascl2 has been shown to work synergistically with β-catenin/Tcf4 to jointly promote the expression of cell stemness characteristics [44]. However, Ascl2 is not an oncogene and its primary role is to regulate intestinal crypt stemness. In Apc^min/+^ mice, overexpression of Ascl2 did not lead to tumor development [45].

#### DCLK1

Doublecortin-like kinase 1 (DCLK1) is another marker of colorectal CSCs. The *DCLK1* gene is located on human chromosome 13, and its upregulation is associated with the prognosis and metastasis of colorectal cancer [46]. Unlike Lgr5, which is shared by normal and tumor stem cells, DCLK1 was shown to be able to distinguish between normal and tumor stem cells in the gut. Taking advantage of *Lgr5*^CreERT2/+^; *Ctnnb1*^lox(ex3)/+^ and *DCLK1*^CreERT2/+^; *Ctnnb1*^lox(ex3)/+^ mice, Nakanishi Y et al. demonstrated that normal intestinal DCLK1^+^ cells did not become CSCs, and DCLK1 expression emerged in Lgr5^+^ CSCs. In addition, they used *DCLK1*^CreERT2/+^; *Rosa26R*; *Apc*^Min/+^; *Rosa26*^iDTR/+^ mice to delete DCLK1^+^ cells, resulted in tumor regression [47]. Westphalen CB et al. used dextran sulfate sodium (DSS) to induce inflammatory activation in *DCLK1-CreERT* × *Apc*^flox/flox^ mice, ultimately leading to tumorigenesis [48]. Chandrakesan P et al. isolated DCLK1^+^ cells from the intestines of *APC*^min/+^ mice and found that the CSC markers Lgr5, Bmi1 and Musashi1 were highly expressed in DCLK1^+^ cells, and the activities of β-catenin, Notch and NF-kB pathways were highly activated, which were all related to the occurrence of intestinal tumors [49]. Several mechanisms of DCLK1 in CSC regulation have been identified. DCLK1 can enhance the expression of PGE2 through the XRCC5/COX2 axis and promote the stemness and invasiveness of CRC [50]. DCLK1 also promotes β-catenin signaling by stabilizing CCAR1, thereby enhancing tumor cell stemness and mediating 5-fluorouracil resistance in CRC [51].

### Signaling pathways

Generally, CSCs share many characteristics with embryonic and normal tissue stem cells, such as activation of the Wnt/β-catenin, Notch, Hedgehog and Hippo/Yap signaling pathways [52]. In colorectal CSCs, the most extensively investigated pathway is the Wnt/β-catenin pathway, whereas the Hippo/Yap1 and JAK-STAT pathways have emerged as new modulators in CSC regulation [53]. Here, we review recent progress in understanding the Wnt/β-catenin, Hippo/Yap, JAK-STAT and Notch signaling pathways.

#### Wnt/β-catenin

**Fig. 1:**
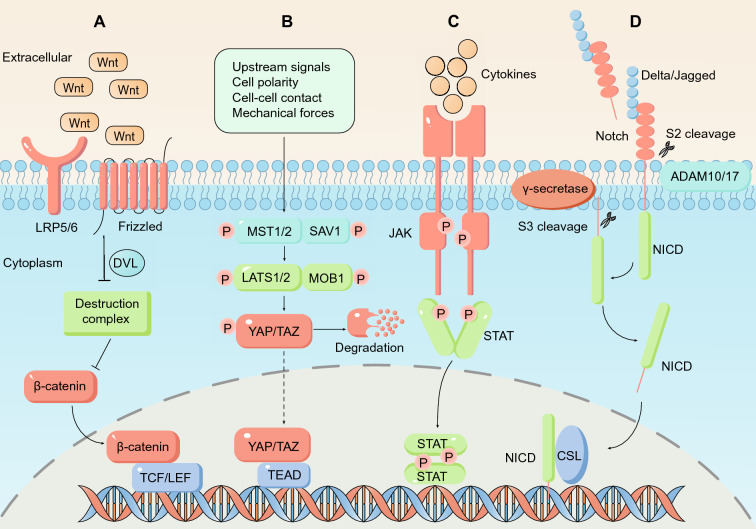
Signaling pathways in colorectal CSCs. **A** Wnt/β-catenin signaling pathway. In the presence of Wnt ligand proteins, the engagement of Wnt, Frizzled and LRP5/6 on the cell surface induces the activation of downstream DVL signaling, which in turn inhibits the β-catenin destruction complex (which is mainly composed of APC, Axin1/2, GSK3β and CK1α), allowing β-catenin to avoid being degraded, to accumulate in the cytoplasm and enter the nucleus. β-catenin enters the nucleus and binds with TCF/LEF to promote the transcription and expression of target genes. **B** Hippo/Yap signaling pathway. Upon stimulation via upstream signaling (mainly cell polarity, cell contact, and mechanical force signals), phosphorylated MST1/2 phosphorylate LATS1/2, and then, activated LATS1/2 phosphorylate YAP/TAZ to induce their degradation. In the absence of upstream signal stimulation, dephosphorylated YAP/TAZ aggregate in the nucleus and combine with the transcription factors TEAD1-4 to promote the expression of target genes. **C** JAK/STAT signaling pathway. When cytokines bind plasma membrane the receptors, receptors dimerize, and receptor-associated JAK kinase is activated via mutual phosphorylation. Then, the activated JAK kinase phosphorylates receptor tyrosine residues and recruits and activates SH2 domain-containing STAT, which dissociates from the receptor, forms dimers in the cytoplasm and enters the nucleus to regulate the expression of target genes. **D** Notch signaling pathway. Delta-like ligands (DLL1, DLL2, DLL3 and DLL4) and Jagged ligands (JAG1 and JAG2) adjacent to cells are ligands for Notch receptors (including Notch 1, Notch 2, Notch 3 and Notch 4). Through a combination of ligands and receptors, the S2 site in Notch receptors is cleaved by the ADAM10 or ADAM17 protease, resulting in the release of the extracellular portion of Notch. Then, γ-secretase cleaves the Notch receptor at the S3 site, allowing the release of the Notch intracellular domain (NICD). The NICD enters the nucleus and interacts with CSL to regulate the expression of downstream target genes

As the canonical Wnt signaling pathway, the Wnt/β-catenin pathway promotes the proliferation and renewal of colorectal CSCs [54]. In the absence of Wnt ligands, the intracellular β-catenin destruction complex (mainly composed of APC, Axin1/2, GSK3β and CK1α) phosphorylates β-catenin. Subsequently, phosphorylated β-catenin can be ubiquitinated by β-Trcp and degraded through the proteasome pathway [55]. However, in the presence of Wnt ligands, their binding to receptor complexes (Frizzled and LRP5/6) on the cell membrane surface disrupts β-catenin destruction complex through downstream DVL. β-catenin is thus protected from degradation and accumulates in the cytoplasm, ultimately entering the nucleus, it binds to T-cell factor/lymphatic enhancer factor (TCF/LEF) to promote the transcription and expression of target genes such as MYC, LGR5, CD44, CCND1, CCL28, IFNG, and CD274 (PD-L1) [56] **(**Fig. 1A**)**.

The Wnt/β-catenin signaling pathway plays an important role in the formation and maintenance of oncospheres, and the downregulation of β-catenin reduces the transcriptional activity of the TCF/LEF complex and inhibits the formation of oncosphere [57]. The mutation of *APC*, which occurs in 85 percent of colorectal cancer patients, is also closely related to the stemness of colorectal cancer and Wnt/β-catenin activation [58, 59]. Normal ISCs with *APC* deletion tend to be transformed into CSCs and promote CRC tumorigenesis [34]. β-catenin can combine with Klf4 to regulate the expression of telomerase, thereby maintaining the long telomeres of colorectal CSCs and avoiding genomic disaster [60]. Moreover, colorectal CSCs promote the epithelial-mesenchymal transition (EMT) through the Wnt/β-catenin pathway and thus promote tumor cell invasion and metastasis [61].

#### Hippo/Yap

The Hippo/Yap pathway consists of a series of conserved kinases that regulate the proliferation, apoptosis, self-renewal and EMT of CSCs [62]. Among these proteins, two kinase complexes, namely, the MST1/2 complex (containing the adapter protein SAV1) and the LATS1/2 complex (containing the adapter protein MOB1), are key factors [63]. When stimulated by upstream signaling, LATS1/2 phosphorylates the transcriptional coactivator YAP/TAZ to inhibit its function [64]. In the absence of upstream signal stimulation, dephosphorylated YAP/TAZ enters the nucleus, binds to the transcription factors TEAD1-4 and promotes the expression of their target genes [65] **(**Fig. 1B**)**.

YAP is required for CRC progression and CSC self-renewal. YAP reprograms Lgr5^+^ stem cells by inhibiting Wnt signal transduction, and early colorectal CSCs with *APC* mutations need YAP to prevent colorectal CSCs from differentiating into Paneth cells [66]. Interestingly, some studies have indicated that activation of Hippo/YAP limited DVL activity to inhibit Wnt/β-catenin activation, leading to the loss of stemness of colorectal CSCs and inhibiting tumor growth [67, 68].

#### JAK/STAT

The JAK/STAT pathway is one of the most important signaling pathways in human body. Over 50 cytokines and growth factors have been demonstrated to function through the JAK/STAT pathway[69]. Reciprocal phosphorylation of receptor-associated JAK is activated when cytokines bind to plasma membrane receptors, and activated JAK then phosphorylates the plasma membrane receptor, allowing it to recruit STAT via SH2 domain [70]. Ultimately, STAT is phosphorylated and activated by JAK, forms a dimer and translocates to the nucleus to regulate the expression of target genes [71] **(**Fig. 1C**)**.

As a critical member of the STAT family, the activation of STAT3 promotes the proliferation of CRC cells [72], and the expression of STAT3 and activated pSTAT3 in colorectal CSCs is higher than that in normal CRC cells [73]. Colorectal CSCs resist radiotherapy and maintain cell stemness through the action of the JAK2/STAT3/CCND2 axis [74]. In addition, colorectal CSCs resist to death-stimulating signals via the IL-4/JAK3/STAT6 axis [75] and harbor an enhanced EMT capacity through the ENC1/JAK2/STAT5 axis [76].

#### Notch

The Notch signaling pathway is also indispensable for the maintenance of colorectal CSCs, where the Notch signaling is 10 to 30 times more active than in normal colorectal cancer cells [77]. Activation of the Notch signaling pathway requires three-step cleavage of Notch receptors by proteases [78]. Notch receptors (including Notch 1, Notch 2, Notch 3, and Notch 4) are first synthesized in the endoplasmic reticulum and subsequently transported to the Golgi apparatus, where the S1 site of Notch receptors is cleaved by the furin-like protease, and then, Notch receptors are transferred to the cell surface [79]. When Notch ligands (including DLL1, DLL2, DLL3, DLL4, Jagged1, Jagged2) of adjacent cells bind to Notch receptors, their S2 site is cleaved by the ADAM10 or ADAM17 protease, and the extracellular domain of the Notch receptor is released. Finally, the S3 site of the Notch receptors is cleaved by γ-secretase protease, and the Notch intracellular domain (NICD) is released. The NICD translocates to the nucleus and interacts with CSL to induce the transcriptional expression of downstream target genes [80] **(**Fig. 1D**)**. The target genes of Notch signaling pathway include Hes family genes [81], GATA3 [82] and c-myc [83].

The expression of Hes1, a target gene of the Notch signaling pathway, can promote the metastasis of CRC.It is related to the poor prognosis of CRC patients [84]. A study showed that miR-195-5p directly bound Notch2, thereby inhibited the stemness of CRC and increased the sensitivity of drug-resistant human CRC SW620 cells to 5-Fu [85]. Another study revealed that serine-threonine kinase receptor-associated protein (STRAP) promoted the stemness of CRC cells through the action of the STRAP/NOTCH1/HES1 signaling axis [86].

### Epigenetic modifications

Epigenetic modifications, which changes chromatin architecture and gene expression without altering the DNA sequence, is emerging as a critical regulatory element in various physiological and pathological processes [87–89], and their roles in CSCs have been extensively explored. Epigenetic modifications, including DNA methylation, histone modification, chromatin remodeling and non-coding RNAs (ncRNAs), modulate multiple important biological processes related to colorectal CSCs, such as the expression of CSC marker expression and the activation of signaling.

#### DNA methylation

As a type of chemical modification, DNA methylation refers to the covalent binding of a methyl group to the 5 'carbon position of the cytosine under the action of DNA methyltransferase, such as Dnmt1, Dnmt3a or Dnmt3b [90]. An increasing number of studies have demonstrated that DNA methylation controls gene expression by altering chromatin structure, DNA conformation, DNA stability and DNA interactomes [91]. Generally, DNA methylation is associated with gene silencing, although its precise function appears to vary according to different genomic contexts, such as the concentration of CpG islands, placement of transcription start sites, and the presence of gene bodies and regulatory elements [92].

**Fig. 2:**
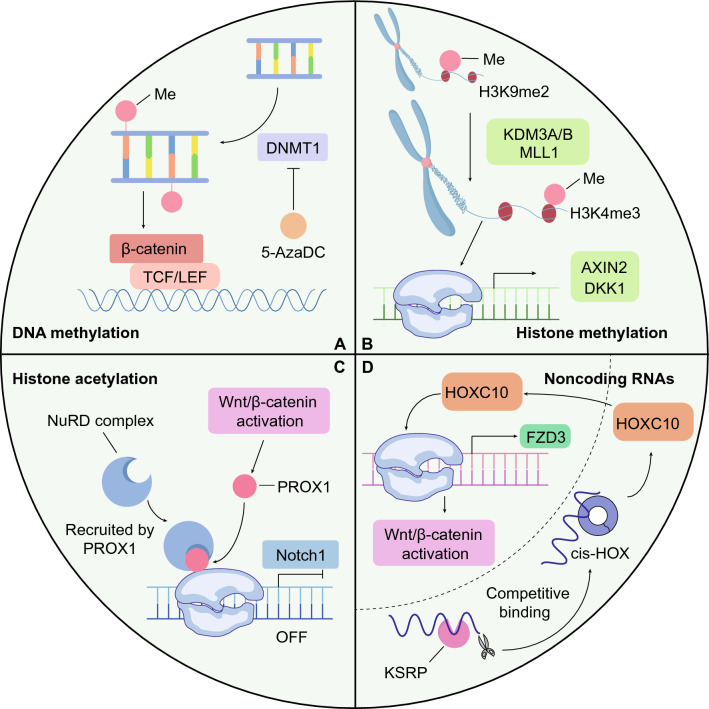
Epigenetic modifications of colorectal CSCs. **A** DNA methylation. DNA methyltransferase 1 (DNMT1) participates in the activation of the Wnt/β-catenin pathway to maintain the stemness of CRC cells. After knocking down DNMT1 or treating cells with the DMNT1 inhibitor 5-aza-2'-deoxycytidine (5-AzaDC), the transcriptional activity of β-catenin in the nucleus was significantly reduced. **B** Histone methylation. Repressive H3K9me2 mark wasmarks are removed by histone demethylases (KDM3A/B), which simultaneously recruit lysine methyltransferase (MLL1), which mediates for the H3K4me3 modification to and thus promotes the expression of the Wnt target genes *AXIN2* and *DKK1*. **C** Histone acetylation. The Wnt/β-catenin target gene PROX1 interacts with the *Notch1* promoter and recruits the nucleosome remodeling and deacetylase (NuRD) complex, which deacetylates histones and remodels chromatin to block *Notch1* transcription. **D** Noncoding RNAs. cis-HOX (a cyclic RNA) can binds to HOXC10 mRNA in the cytoplasm to inhibit the KSRP-dependent degradation of HOXC10 mRNA. The increased HOXC10, the level of which has been increased, enters the nucleus and drives *FZD3* expression to activate the Wnt/β-catenin signaling pathway

Typically, DNA methyltransferases are highly expressed in various tumor cells, and DNA methylation has been closely associated with depletion of tumor suppressors and differentiation genes [93]. Accordingly, DNA methyltransferases have emerged as novel targets for tumor prevention [94]. *DNMT1* knockout HCT116 cells exhibited decreased CSC marker expression and impaired tumor-initiating ability but a growth rate similar to that of DNMT1-WT cells, indicating the involvement of DNMT1 in CSC-specific maintenance and functionality [95]. Interestingly, another work revealed that DNMT1 regulated the activation of the Wnt/β-catenin signaling pathway, the most important regulatory signaling pathway in CSCs. *Dnmt1* knockout decreased the total β-catenin level and blocked the nuclear translocation of β-catenin [96]. Similarly, the DNMT inhibitor 5-Aza-2′-deoxycytidine (5-AzaDC) significantly reduced CSC maintenance and inhibited the activation of β-catenin **(**Fig. 2A**)**. Mechanistically, the promoter regions of Wnt/β-catenin inhibitory genes are frequently methylated in CRC cells and CSCs, leading to their decreased expression and subsequent activation of the Wnt/β-catenin pathway [97].

#### Histone modifications and chromatin remodeling

Histone modifications, including mainly methylation and acetylation, occur at various sites of histones, mainly at lysine and arginine residues. Histone methylation results in transcription inhibition or activation of corresponding genes, depending on the position of the modified amino acid residues, the extent of the modification and the nature of the methyltransferase [98, 99]. In contrast, histone acetylation generally induces transcriptional activation [100]. Histone modification regulates chromatin structure through multiple mechanisms, and the dynamic regulation of chromatin results in chromatin remodeling [101]. As critical layers of epigenetic regulation, histone modifications and chromatin remodeling are intricately involved in CSC modulation.

The regulation of typical marker of transcriptional activation, histone 3 trimethylated on lysine 4 (H3K4me3) is mediated by histone demethylase KDM3 and lysine methyltransferase KMT2A (also named MLL1). Through short interfering RNA (siRNA) screening, KDM3A and KDM3B were identified as regulators of Wnt/β-catenin activation and CSC maintenance. Interestingly, KDM3A/B promoted the transcription of the Wnt/β-catenin target genes AXIN2 and DKK1 through erasing H3K9me2 marks and promoting MLL1-dependant H3K4me3 [102] **(**Fig. 2B**)**. H3K4me3 remodels chromatin and drives transcription by promoting the interaction of the marked sequence with the nucleosome remodeling factor (NuRF) complex [103], which is also required for the self-renewal of CSCs in various tumors, including liver cancer [104], gastric cancer [105] and CRC [106]. For colorectal CSCs, the NURF complex is recruited onto the promoter of EHF, a core transcription factor for Lgr4/5 expression and CSC self-renewal [106].

Histone acetylation is also involved in CSC self-renewal and tumorigenesis control. In colorectal CSCs, the Wnt/β-catenin target gene PROX1 is highly expressed, and Notch inhibition increases the number of PROX1-positive CSCs. In contrast, PROX1 inhibits the promoter activation and transcription of *Notch1* in a nucleosome remodeling and deacetylase (NuRD) complex-dependent manner. PROX1 interacts with and recruits the NuRD complex to the *Notch1* promoter, through which NuRD drives histone deacetylation and chromatin remodeling to ultimately block the transcription of *Notch1* [107] **(**Fig. 2C**)**. Interestingly, the NuRD complex functions in niche cells to modulate CSC activity. For example, enteric serotonergic neurons secrete 5-HT to drive the self-renewal of colorectal CSCs, and the production of 5-HT is regulated by the NuRD complex [108].

In addition to the NuRF and NuRD complexes, other nucleosome-remodeling complexes, such as the SWI/SNF, TIP60/P400 and PRC2 complexes, are highly abundant and activated in various CSCs, collectively confirming aberrant epigenetic regulation in tumor tumorigenesis and CSC maintenance [109–111].

#### Noncoding RNAs

NcRNAs, including microRNAs (miRNAs), long noncoding RNAs (lncRNAs), and circular RNAs (circRNAs), have emerged as central regulators in various physiological and pathological processes, including tumorigenesis [112]. The regulatory roles of ncRNAs in CSCs and niche cells have been extensively discussed in another review [113], and therefore, here, we exclusively focus on several ncRNAs involved in Wnt/β-catenin activation and CSC metabolism.

The Wnt/β-catenin signaling pathway is activated in CSCs and is the most important pathway involved in CSC self-renewal; however, the mechanism underlying the initiation of Wnt/β-catenin signaling has been rarely investigated. In colorectal CSCs without *APC* mutations, Wnt/β-catenin is initiated by FZD3, a Wnt receptor highly expressed in colorectal CSCs. Moreover, cis-HOX drives FZD3 expression in a HOXC10-dependent manner [114] **(**Fig. 2D**)**. Interestingly, FZD6, another Wnt receptor, is highly expressed in liver CSCs. Mechanistically, the FZD6 promoter is activated by lncFZD6, which is also highly expressed in CSCs [115]. The divergent Wnt receptors and initiating mechanism of Wnt/β-catenin in colorectal and liver CSCs demonstrate intertumoral heterogeneity in Wnt/β-catenin initiation.

As hallmarks of tumors, metabolic disorders are precisely regulated by ncRNAs [116]. Compared with non-CSCs and normal cells, glycolysis is activated and oxidative phosphorylation (OXPHOS) is inhibited in CSCs. mcPGK1, which is coded by mitochondrial DNA, is highly expressed in CSCs and drive the metabolic switch. mcPGK1, which is encoded by mitochondrial DNA, is highly expressed in CSCs and drives metabolism switching. mcPGK1 promotes the interaction of PGK1 and the TOMM40 complex, facilitates PGK1 entry into mitochondria and ultimately inhibits OXPHOS in a PGK1-dependent manner [117]. In addition to glucose metabolism, lipid metabolism is regulated by ncRNAs in CSCs. LncROPM (a regulator of phospholipid metabolism) is highly expressed in CSCs, enhances the stability of PLA2G16 mRNA, and ultimately promotes phospholipid metabolism and arachidonic acid production. Arachidonic acid, in turn, activates Wnt/β-catenin and Hippo/YAP signaling in CSCs [118]. Accordingly, ncRNAs have emerged as critical metabolic modulators in CSC regulation.

## Colorectal CSCs and the immune niche

The regulation of self-renewal of CSCs and activation of CSC-related signaling pathways is fine tune by various intracellular and extracellular factors. Because of the great success and promising prospects of immunotherapy for patients with hematological malignancies and solid tumors [119, 120], the immune niche of CSCs has attracted more attention [121]. In this section, we review recent progress in investigating the mutual interaction between colorectal CSCs and their immune niches.

### Expression of immune proteins on colorectal CSCs

Since immune checkpoint inhibitors (ICIs) have proven effective in eliminating melanoma and other tumors [122], targeting immune checkpoints has become one of the main strategies of current immunotherapy. However, similar to that CSCs in many other tumors, the crosstalk between colorectal CSCs and immune cells shapes an inhibitory immune microenvironment and promotes tumor progression [9]. This is consistent with the current situation that CRC patients are difficult to benefit from anti-PD-1/PD-L1 therapy [123]. However, immunotherapy exerts an effect on some subsets of colorectal tumors. In fact, the expression levels of the immune markers FoxP3, PD-L1 and CD3 greatly affect the prognostic value of the CSC markers SOX2 and CD133, indicating the close relationship between CSCs and antitumor immunity [124]. Recently, an increasing number of studies have revealed that many immune-associated proteins are expressed on nonimmune cells, accounting for the immunological regulatory function of nonimmune cells [125]. Therefore, the expression profile of immune-related surface proteins on colorectal CSCs is helpful for understanding the interaction between colorectal stem cells and niche immune cells.

#### Low MHC expression on colorectal CSCs

**Fig. 3:**
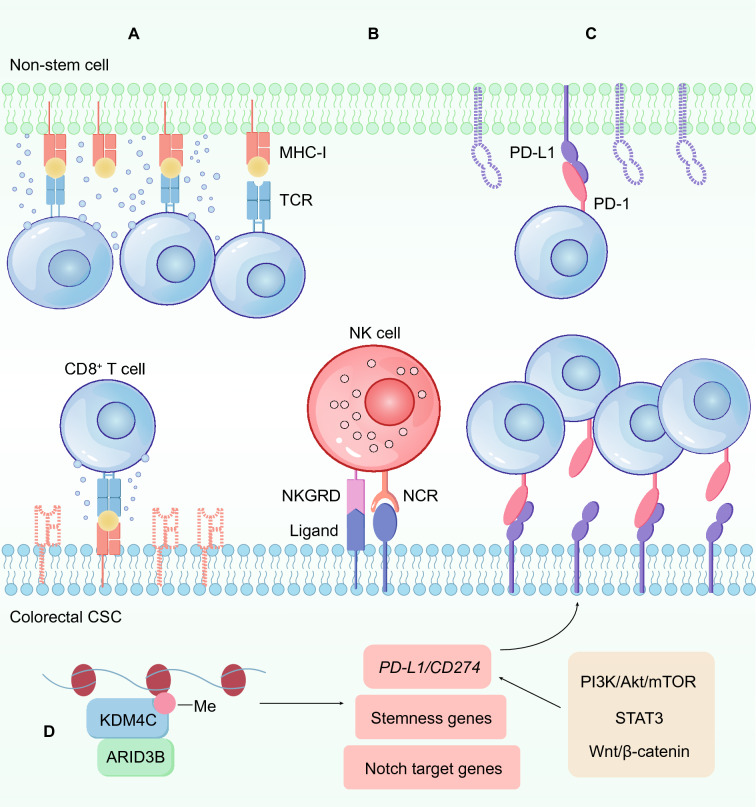
Expression profiles of immune proteins on colorectal CSCs. **A** The expression of MHC class I molecules on colorectal CSCs is downregulated, making them difficult to recognize and kill by CD8^+^ T cells. **B** NK cells are activated by receptors such as NKG2D and natural cytotoxicity receptors (NCRs), which recognize and kill CSCs in an MHC-independent manner. **C** Colorectal CSCs express high levels of the immune checkpoint PD-L1, which can inhibit the antitumor immune effect of T cells. **D** *PD-L1* and colorectal CSC-related genes are downstream of the same transcriptional element. The ARID3B-KDM4C complex regulates chromatin state to activate downstream Notch target genes, CRC stemness genes and *PD-L1* transcriptional expression. In addition, *PD-L1* is the direct target gene of the Wnt/β-catenin, PI3K/Akt/mTOR, and STAT3 signaling pathways

Among all subsets of immune cells, the CD8^+^ T cells play a primary role in the antitumor immune effects [126]. A neoantigen peptide mutated in tumor cells can be presented at the cell surface of MHC-I class molecules, and the T-cell receptor (TCR) on the surface of CD8^+^ T cells recognizes and binds the neoantigen peptide–MHC complex, thereby exerting an antitumor immune effect by killing tumor cells directly [127]. However, the expression of MHC class I molecules is downregulated in colorectal CSCs [128], which limits the recognition and tumor-killing effect of CD8^+^ T cells [129] **(**Fig. 3A**)**.

In contrast to CD8^+^ T cells, natural killer (NK) cells, constituting another subset of killer cells in the immune system, can recognize and kill MHC-I-low cells in an MHC-independent manner. Notably, colorectal CSCs express ligands, including NKG2D, natural cytotoxicity receptors and DNAM-1, that lead to NK activation [130] **(**Fig. 3B**)**. Therefore, NK-based immunotherapy is expected to become a new method for the treatment of colorectal cancer and CSCs [131]. Unfortunately, another study revealed that CRC cells escape NK killing because of downregulated NKG2DL, indicating that further investigation is needed to develop better methods to kill CRC cells and CSCs via NK cells [132].

#### High expression of immune checkpoint molecules

PD-L1 is the most extensively characterized immune checkpoint on the surface of tumor cells, and immunotherapy targeting PD-1/PD-L1 has opened a new era of antitumor therapy [133, 134]. When the ligand PD-L1 binds to PD-1 on the surface of T cells as a ligand, the activation of T cells activation is impaired inhibited. On the one hand, the immune receptor tyrosine–based inhibitory motif (ITIM) and immune receptor tyrosine–based switch motif (ITSM) at in the cytoplasmic tail of PD-1 are phosphorylated, and then, Src homology region region-2 domain-containing phosphatase (SHP-2) is recruited and activated, and finallyultimately, the activation of TCR and CD28 proximal downstream signaling areis blocked [135]. On the other hand, PD-1 disrupts TCR-pMHC-CD8 trimolecular interactions and directly inhibits the antigen recognition process of T cells [136]. Collectively, PD-L1 plays a significant inhibitory role in antitumor immunity.

Accumulating evidence has revealed that colorectal CSCs evade immune surveillance by hijacking the PD-L1 antitumor pathway, and previous studies have demonstrated that PD-L1 is highly expressed in colorectal CSCs [137, 138] **(**Fig. 3C**)**. Mechanistically, the upregulation of PD-L1 in colorectal CSCs is induced mainly via two pathways **(**Fig. 3D**)**. On the one hand, PD-L1 and CSC-related genes are activated downstream of the same transcriptional element. For example, colorectal CSCs express high levels of ARID3B, and ARID3B binds with KDM4C to form a complex, which directly binds to the regulatory region of PD-L1, as well as activates Notch target genes and stem-related genes. Thus, PD-L1 is coexpressed with Notch target genes in colorectal CSCs [139]. On the other hand, PD-L1 is a direct target gene of critical pathways involved in CSC maintenance, including the PI3K/Akt/mTOR, STAT3 and Wnt/β-catenin pathways. Insulin stimulates the expression of PD-L1 in colorectal CSCs through the PI3K/Akt/mTOR signaling pathway, and EGF further enhances the stability of PD-L1 on the cell membrane [140]. Moreover, colorectal CSCs express low levels of S100A14, which initiates the high expression of PD-L1 by affecting the stability of STAT3 [141]. Recently, an increasing number of studies have proven that PD-L1 is directly regulated by the canonical Wnt/β-catenin signaling pathway. In *APC* mutant CRC cells, overexpressed β-catenin formed a complex with TCF4, which bound the promoter region of the PD-L1-encoding gene, to promote the transcription of PD-L1, indicating that PD-L1 is a direct target of β-catenin [142]. Another study demonstrated that PD-L1 and other immune checkpoint molecules, including TIM3 and CD24, were direct target genes of Wnt/β-catenin pathways, and collectively these immune checkpoint molecules accounted for the immune escape of CSCs [143].

In addition to PD-L1, CD-47, another immune checkpoint involved in the macrophage phagocytosis is also expressed on CSCs, and it promotes the malignancy of EMT-associated CRC cells and enhances the stemness of CRC cells [144].

### CSC–immune cell crosstalk during tumorigenesis

Tumorigenesis is a complicated process, and the biogenesis and expansion of cancer CSCs are typical events in the early stage of tumorigenesis. Ultimately, tumorigenesis initiation requires stable inheritance of oncogenic variants that enable to outcompete neighboring normal cells to gain advantages and the clonal expansion of CSCs, escape of CSCs from immune surveillance, and remodeling of the immune microenvironment [145]. Here, we mainly summarize the recent progress in the latter two CSC advantages.

#### Colorectal CSCs escape immune surveillance

**Fig. 4:**
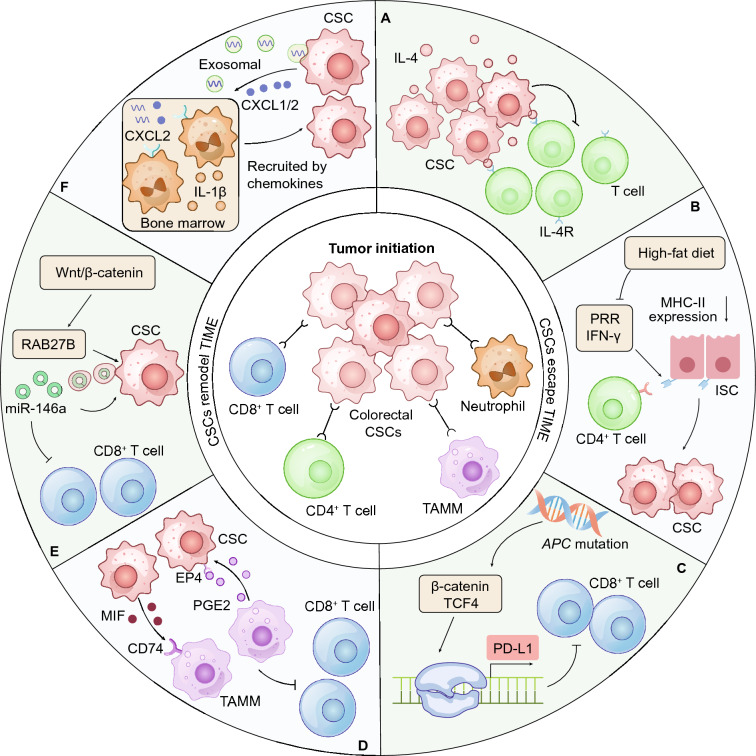
CSC-immune cell crosstalk involvement in tumor initiation. **A** Colorectal CSCs suppress the proliferation of T cells via high expression of IL-4. **B** A high-fat diet reduced the expression of MHC class II molecules in colorectal CSCs by inhibiting the PRR and IFNγ signaling pathways, thereby inhibiting the activity of CD4^+^ T cells and promoting CRC occurrence. **C** *APC* mutation, the major oncogenic variant in early colorectal tumorigenesis, activates the Wnt/β-catenin signaling pathway, and the β-catenin-TCF4 complex binds to the promoter of the PD-L1-encoding gene to induce PD-L1 expression, which inhibits CD8^+^ T cell activation. **D** Colorectal CSCs secrete macrophage migration inhibitory factor (MIF), which binds the CD74 receptor on tumor-associated monocytes and macrophages (TAMMs), inducing immunosuppressive signaling. In addition, TAMMs secrete prostaglandin E2 (PGE2) and promote the proliferation of colorectal CSCs via the action of the PGE2 receptor EP4. **E** The Wnt/β-catenin signaling pathway activates the expression of RAB27B to mediate the secretion of miR-146a exosomes, which promote the stemness of tumor cells and tumorigenicity and reduces the infiltration of CD8^+^ T cells. (**F**) Colorectal CSCs secrete exosomal RNAs to increase the number of neutrophils in the bone marrow and induce neutrophils to express IL-1β. In addition, colorectal CSCs secrete CXCL1/2 to recruit neutrophils expressing CXCR2 in bone marrow, and IL-1β secreted by neutrophils can promote tumorigenesis

The surveillance function of the immune system can identify and eliminate cancer cells and prevent the occurrence of early cancers. However, colorectal CSCs can evade immune surveillance and initiate tumorigenesis in multiple ways. A study demonstrated that colorectal CSCs suppressed the proliferation of T cells by secreting high levels of IL-4 secretion. In a coculture system of colorectal CSCs, peripheral blood mononuclear cells (PBMCs), and phytohemagglutinin (PHA)/ concanavalin (Con) A, the proliferation and activation of T cells were inhibited by colorectal CSCs [128] **(**Fig. 4A**)**. A high-fat diet, a risk factor for colorectal tumorigenesis, can induce the downregulation of MHC-II molecules in colorectal CSCs, thereby inhibiting the direct or indirect killing effect of CD4^+^ T cells. In mice, APC^null^ MHC-II^−^ colorectal CSCs were more tumorigenic than APC^null^ MHC-II^+^ colorectal CSCs [146] **(**Fig. 4B**)**. In addition, PD-L1 and CD47 on the surface of colorectal CSCs also led to early escape of tumor cells from immune surveillance during tumor development. The *APC* mutation, the major oncogenic variation in early colorectal tumorigenesis, also promotes the expression of the immune checkpoint PD-L1 via Wnt/β-catenin signaling, drives the immune resistance to CD8^+^ T cells, and ultimately initiates colorectal tumorigenesis [142] **(**Fig. 4C**)**.

#### Colorectal CSCs remodel the immunosuppressive microenvironment

In the early stage of CRC carcinogenesis, colorectal CSCs remodel the immune microenvironment, undergo immune escape and drive their own self-propagation Exploiting DNA label retention, single-cell RNA sequencing, coculturing, cell depletion assays, and lineage tracing, He et al. revealed that intestinal CSCs remodel the tumor microenvironment (TME); for example, they remodel tumor-associated monocytes and macrophages (TAMMs), which in turn promote CSCs through the PGE2-EP4 pathway. In general, the crosstalk between CSCs and TAMMs drives the formation of an immunosuppressive and protumorigenic niche [147] **(**Fig. 4D**)**. Moreover, colorectal CSCs secrete miR-146a-loaded exosomes, which in turn reduce the infiltration rate of CD8^+^ T cells in tumors and thus promote the formation of an immunosuppressive microenvironment [148] **(**Fig. 4E**)**. In addition, exosomal RNAs secreted by colorectal CSCs expanded the neutrophil population in bone marrow and induced the neutrophils to secrete IL-1β. Moreover, colorectal CSCs secreted CXCL1/2, which recruited CXCR2-expressing neutrophils from the bone marrow to the tumor niche. Reciprocally, IL-1β secreted by these neutrophils promoted tumorigenesis and CSC self-renewal [149] **(**Fig. 4F**)**. Interestingly, these neutrophils also inhibited the proliferation and function of T cells by reducing the expression of IL-2, which is a key mediator of T-cell activation [150] and antitumor effects [151].

### CSC-immune cell crosstalk in tumor maintenance

The crosstalk between CSCs and immune cells is involved not only in tumor initiation but also in tumor maintenance. In established tumors, CSC self-renewal and differentiation are regulated by CSC-intrinsic immune factors and extrinsic immune cells. In addition, CSCs reshape the immune microenvironment, ultimately promoting the maintenance of tumors and CSCs.

#### CSC-intrinsic immune factors.

**Fig. 5:**
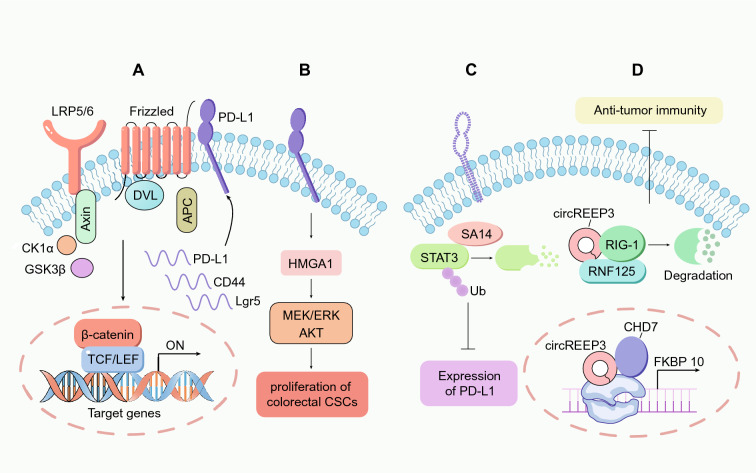
CSC-intrinsic immune factors in CSC-immune cell crosstalk. **A** PD-L1 interacts with Frizzled 6 to inhibit the degradation of β-catenin via the destruction complex. Moreover, the activation of the Wnt/β-catenin pathway induces the expression of PD-L1, forming a positive feedback β-catenin/PD-L1 loop to promote the stemness and expansion of colorectal CSCs. **B** PD-L1 interacts with the HMGA1 to promote the proliferation of colorectal CSCs by activating the HMGA1-dependent MEK/ERK and AKT signaling pathways. **C** Colorectal CSCs express low levels of S100A14 (SA14) to avoid the degradation of STAT3 in CSCs, and thus PD-L1 is highly expressed as a target of STAT3. **D** circREEP3 recruits the chromatin-remodeling protein CHD7 to the promoter of FKBP10 to activate its transcription and promote tumor progression. In addition, circREEP3 can enhance the ubiquitination and degradation of RIG-1 mediated by RNF125, thereby inhibiting antitumor immune effects

As described in the previous section, some immune checkpoint molecules are highly expressed in CSCs and are active through various pathways, and more importantly, these immune checkpoint molecules are key modulators in CSC function. On the surface of colorectal CSCs, PD-L1 interacts with Frizzled6 (FZD6) to activate β-catenin, thereby promoting the expression of Wnt/β-catenin target genes and driving the stemness of colorectal CSCs. Interestingly, PD-L1 is also a target of the Wnt/β-catenin pathway, and the positive feedback of PD-L1-Wnt/β-catenin highlights CSC–immune cell cross-talk [152] **(**Fig. 5A**)**. Moreover, PD-L1 interacts with HMGA1 to promote the proliferation of colorectal CSCs through the PI3K/Akt and MEK/ERK pathways [153] **(**Fig. 5B**)**. Colorectal CSCs with high PD-L1 expression showed an enhanced EMT phenotype, suggesting that PD-L1 also regulates the invasion capacity of colorectal CSCs [138].

In addition to immune checkpoint molecules, some other immune-associated factors also modulate the self-renewal of colorectal CSCs. S100A14 interacts with and induces the degradation of STAT3 and thus impairs PD-L1 expression and the tumorigenesis and chemoresistance of colorectal CSCs [141] **(**Fig. 5C**)**. CircREEP3, a circular transcript involved in RIG-1-dependent antitumor immunity, drives the tumorigenic, metastatic and stem cell-like phenotypes of CRC cells, further indicating crosstalk between innate immune cells and CSCs [154] **(**Fig. 5D**)**.

#### The immune microenvironment regulates colorectal CSCs

**Fig. 6:**
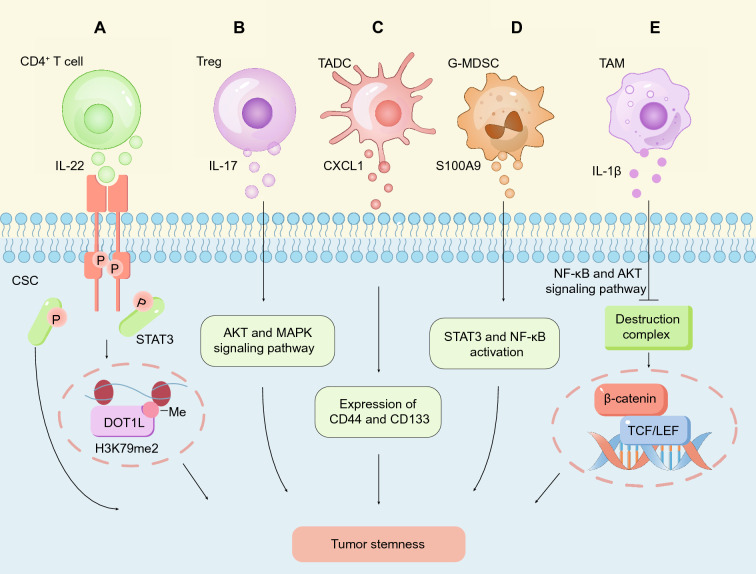
Immune microenvironment regulates colorectal CSCs to drive tumor stemness. **A** CD4 + T cells secrete IL-22, which activates STAT3 in colorectal CSCs. The activation of STAT3 promotes the stemness of CRC and induces the expression of DOT1L, which promotes the expression of stemness-related genes by promoting H3K79me2 deposition at their promoter regions. **B** Treg cells act on colorectal CSCs by secreting IL-17 and promote the maintenance of CRC stemness through the AKT and MAPK signaling pathways. **C** CXCL1 secreted by tumor-associated dendritic cells (TADCs) promotes the expression of CD44 and CD133 and maintains the stemness of CRC. **D** Granulocyte myeloid-derived suppressor cells (G-MDSCs) secrete S100A9-containing exosomes that promote the stemness of colorectal CSCs by inducing STAT3 phosphorylation and NF-κB activation. **E** IL-1β secreted by tumor-associated macrophages (TAMs) inhibits the phosphorylation activation of GSK3β by activating the NF-κB and AKT signaling pathways, thereby abrogating the β-catenin destruction complex and activating the Wnt/β-catenin signaling pathway in colorectal CSCs

As the key immune cells in antitumor function, T cells profoundly modulate the self-renewal of CSCs. The Weiping Zou laboratory team identified IL-22^+^CD4^+^ T cells in CRC tissues, and these cells were recruited via the CCR6-CCL20 axis. Moreover, IL-22^+^CD4^+^ T cells secreted IL-22, and IL-22 in turn promoted the stemness of CRC cells via STAT3 activation and the expression of the H3K79 methyltransferase DOT1L [155] **(**Fig. 6A**)**. Interestingly, a recent study also revealed that iNKT17-derived IL-22 promoted the liver metastasis of CRC by facilitating cancer cell extravasation in an endothelial cell-dependent manner [156]. Regulatory T cells (Tregs), a major subset of T cells involved in immune suppression, suppressed the antitumor activity of CD4^+^ T cells and CD8^+^ T cells in the tumor immune microenvironment [157]. The level of Tregs was measurable detectable in CRC tissues, and these Tregs secreted IL-17 to enhance the stemness of colorectal CSCs through AKT and MAPK signaling [158] **(**Fig. 6B**)**.

Recently, myeloid cells have emerged as critical modulators in the tumor immune microenvironment, and their roles in CSC regulation and tumor targeting have been extensively investigated [159]. Tumor-associated dendritic cells (TADCs) secreted CXCL1, which in turn increased the expression of the CSC markers CD44 and CD133 on the SW620 CRC cell line and promoted their stemness **(**Fig. 6C**)**. In addition, CXCL1 increased the invasion and EMT rates of SW620 cells [160]. Constituting a type of myeloid-derived suppressor cells (MDSCs) [161], granulocyte myeloid-derived suppressor cells (G-MDSCs) secrete S100A9-containing exosomes to enhance the stemness of CRC cells. CRC cells treated with S100A9 produced more ROS and Nox4, which enhanced the stemness of CSCs by inducing the phosphorylation levels of STAT3 and NF-κB p65 [162] **(**Fig. 6D**)**. As an important component of the tumor immune microenvironment [163], tumor-associated macrophages (TAMs) explain the interaction between CSCs and immune cells. IL-1β secreted by TAMs inactivates GSK3β phosphorylation by activating the NF-κB and AKT signaling pathways, thereby abrogating the β-catenin destruction complex and activating the Wnt/β-catenin signaling pathway in CRC cells [164] **(**Fig. 6E**)**. In addition, as a potential marker of type 2 TAMs (M2-TAMs), JMJD8 expression has been positively correlated with CRC stemness maintenance, chemotherapy resistance and immunosuppression [165].

#### Colorectal CSCs remodel the immune microenvironment

**Fig. 7:**
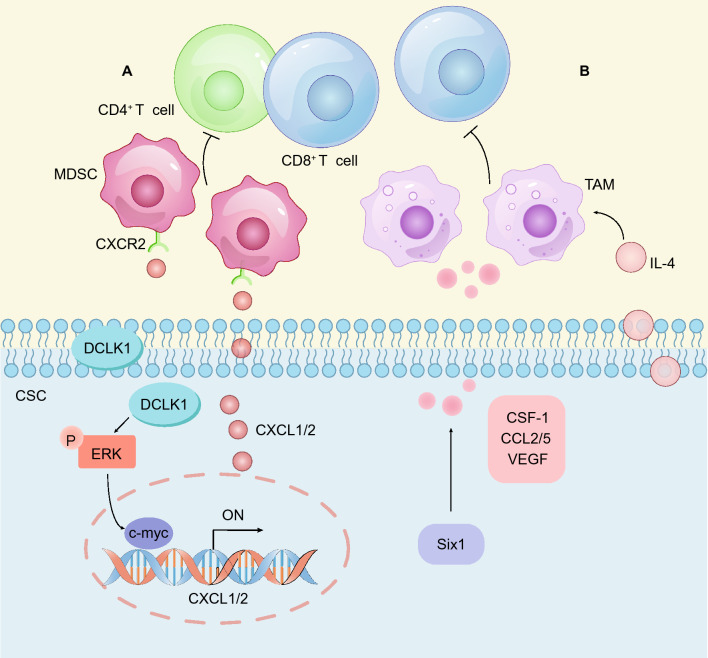
Colorectal CSCs remodel the immune microenvironment. **A** DCLK1 expressed by colorectal CSCs activates the ERK signaling pathway and promotes the expression of CXCL1/CXCL2 to recruit myeloid-derived suppressor cells (MDSCs), thereby inhibiting the activity of CD4^+^ T cells and CD8^+^ T cells. **B** Six1 expressed by colorectal CSCs promotes the secretion of CSF-1, CCL2/5 and VEGF and recruits TAMs to form an immunosuppressive microenvironment. IL-4 secreted by colorectal CSCs is an important factor for maintaining TAM tumor-promoting activity

As a potential marker of CSCs in CRC [47], DCLK1 is involved in remodeling the immune microenvironment. DCLK1 expression has been significantly and positively correlated with the infiltration of various immune cells, including TAMs and Tregs, and is associated with M2-TAM polarization and T-cell exhaustion [166]. In addition, DCLK1 promotes the expression of CXCL1/CXCL2 via the ERK signaling pathway. Subsequently, CXCL1/CXCL2 recruits MDSCs, which suppress the activity of tumor-specific T cells [167] **(**Fig. 7A**)**. Six1 is another candidate marker of colorectal CSCs. Six1-overexpressing CRC cells showed enhanced stemness, and Six1 increased the secretion levels of CSF-1, CCL2/5 and VEGF, thereby recruiting more TAMs that induce immunosuppression [168] **(**Fig. 7B**)**.

In addition to the recruitment of immune cells, colorectal CSCs secrete cytokines and chemokines to remodel the TME in established tumors, and among these cytokines, interleukin-4 (IL-4) has been intensively studied. CD133^+^ colorectal CSCs secrete high levels of IL-4; IL-4/IL-4R blockers promote the antitumor effects of chemotherapeutic drugs, while IL-4 promotes the survival of colon CSCs [25]. Mechanistically, IL-4 activates the STAT6 signaling pathway to promote the expression of BIRC5, a well-characterized apoptosis-inhibitory protein [75]. In addition, IL-4 is required for the activity of cathepsins in TAMs and accounts for the tumor-promoting function of TAMs [169] **(**Fig. 7B**)**. Overall, colorectal CSCs secrete functional cytokines and chemokines to remodel the TME in established tumors.

### CSC-immune cell crosstalk in tumor metastasis

Tumor metastasis is a major cause of tumor-related death, and the molecular mechanism and targeting strategy have been described in another review [170]. Colorectal CSCs and immune cells are both intricately involved in metastasis. The prion protein (PrPc) is highly expressed in a subset of CD44^+^ colorectal CSCs, and PrPc^+^CD44^+^ colorectal CSCs exhibited enhanced metastatic capacity [20]. Conversely, metastasis regulates the self-renewal of CSCs. SNAIL, a key modulator of tumor metastasis, drives the self-renewal and tumorigenicity of colorectal CSCs through IL-8 and JUN expression [171]. Moreover, metastasis is regulated by immune cells, especially in the formation of the premetastatic niche [172]. MDSCs accumulate in the liver before CRC metastasizes to the liver and promote the survival and clonal expansion of metastatic tumor cells. Moreover, VEGF and the CXCL1-CXCR2 axis are necessary for the formation of the premetastatic niche. VEGF secreted by cells in a colorectal tumor induces TAMs to secrete CXCL1, which recruits CXCR2-expressing MDSCs to the liver, ultimately inducing an immunosuppressive premetastatic niche [173]. These works highlight the close relationship among CSCs and metastatic and immune-regulating factors.

Moreover, during tumor metastasis, CSCs are closely related to immune cells, and their interactions extensively regulate metastasis. As a critical pathway in immune regulation, the TDO2-AHR signaling pathway is activated in colorectal CSCs during metastasis. The TDO2-AHR signaling pathway promotes the liver metastasis of CRC, which requires the suppression of immunosurveillance, and this process is related to the positive regulation of AHR in PD-L1 expression [174]. Another study revealed that colorectal CSCs can fuse with monocytes to form CD45^+^CD14^+^EpCAM^+^ tumor hybrid cells (THCs), which exhibit higher metastatic and immune escape capacities [175]. Collectively, colorectal CSC–immune cell crosstalk affects tumor metastasis.

### CSC-immune cell crosstalk in drug resistance

Drug resistance and relapse are still the direct reasons for the low survival rate of cancer patients [176]. CSCs have been proven to be critical to drug resistance [177]. In multiple types of tumors, CSCs are enriched in a residual cell population after conventional chemoradiotherapy [178]. CSCs protect themselves from being killed by antitumor drugs via the EMT [179], metabolic reprogramming[180], epigenetic modifications [181], autophagy [182] and the DNA damage response [183]. The critical role of colorectal CSCs in chemotherapy resistance has been described in another review [184]. 5-Fu is a main drug for the treatment of CRC patients, and 5-Fu drug-resistant CRC cells highly express CSC markers and maintain cell quiescence through the c-Yes/YAP axis [185].

In addition to colorectal CSCs involvement in drug resistance, the immune microenvironment is involved. TAMs secrete MFG-E8 to activate the STAT3 and Hedgehog signaling pathways in colorectal CSCs and promote the chemotherapy resistance and relapse of colorectal CSCs [186]. Moreover, accumulating evidence has proven that CSCs become resistant to immunotherapy via various mechanisms, including low MHC expression, high expression of immune checkpoint molecules such as PD-L1, low neoantigen production and the formation of an immunosuppressive niche [8].

## Targeting colorectal CSCs

In the past ten years, the advent of immunotherapy, such as ICIs and chimeric antigen receptor (CAR)-T cells, has subverted the traditional antitumor therapy. ICIs have been applied to the treatment of hematological tumors and various solid tumors, and have shown great clinical effects [187]. CSCs harbor the characteristics of self-renewal, tumor initiation, metastasis, and are difficult to be eliminated by traditional antitumor treatments. The elimination of CSCs has always been an important goal of basic research and clinical applications [188]. Notably, research targeting colorectal CSCs through antibodies, tumor vaccines, adoptive cell therapy and small molecule inhibitors has been explored recently in the context of CRC, and the results are reviewed herein.

### Monoclonal antibodies (mAbs)

Since rituximab, the first mAb drug targeting CD20, was approved by the FDA in 1997, mAbs have been shown to be very successful antitumor immunotherapies in clinical practice [189]. New mAbs targeting colorectal CSCs, especially those targeting surface markers and key signaling pathways, are constantly being developed and clinically evaluated. The CD44, CD133, Lgr5 and Notch pathways are frequently targeted to eliminate colorectal CSCs **(**Table 1**)**, whereas the Wnt/β-catenin pathway is relatively rarely investigated, perhaps because of the constitutive activation of Wnt/β-catenin induced by APC dysfunction in approximately 85% of CRC patients.

**Table 1:** Elimination of colorectal CSCs based on monoclonal antibodies

Target	Drugs	Clinical trial title	References
CD44	RG7356	A Study of RO5429083 in Patients With Metastatic and/or Locally Advanced, CD44-Expressing, Malignant Solid Tumors (ID: NCT01358903)	[191]
Notch1	Brontictuzumab	A Dose Escalation Study of OMP-52M51 in Subjects With Solid Tumors (ID: NCT01778439)	[203]
Notch2, Notch3	Tarextumab	A Dose Escalation Study of OMP-59R5 in Subjects With Solid Tumors (ID: NCT01277146)	[205]
Target	Drugs	Action	References
DLL4	Anti-DLL4 mAb	In vivo and in vitro experiments show antitumor activity Combination with chemotherapy drugs increases drug sensitivity	[207, 208]
Carbohydrate epitope	CC188	Recognizing a carbohydrate epitope on the surface of colorectal CSCs inhibits tumor invasion in human colorectal cancer cells	[209]
Progastrin	Hz8CV2	Inhibition of Wnt signaling and self-renewal of colorectal CSCs Combined with chemotherapy drugs to increase drug sensitivity	[211]
Lgr5	BNC101	Antitumor activity against colorectal cancer patient-derived xenograft mice Combination therapy increases efficacy of anti-PD-1 therapy	[193–195]
RSPO3	Anti-RSPO3 mAb	Inhibition of colorectal cancer in patient-derived xenograft mice Inhibition of Wnt target gene expression disrupts cancer stem cell function	[198]

Target	Drugs	Action	References
DLL4	Anti-DLL4 mAb	In vivo and in vitro experiments show antitumor activity Combination with chemotherapy drugs increases drug sensitivity	[207, 208]
Carbohydrate epitope	CC188	Recognizing a carbohydrate epitope on the surface of colorectal CSCs inhibits tumor invasion in human colorectal cancer cells	[209]
Progastrin	Hz8CV2	Inhibition of Wnt signaling and self-renewal of colorectal CSCs Combined with chemotherapy drugs to increase drug sensitivity	[211]
Lgr5	BNC101	Antitumor activity against colorectal cancer patient-derived xenograft mice Combination therapy increases efficacy of anti-PD-1 therapy	[193–195]
RSPO3	Anti-RSPO3 mAb	Inhibition of colorectal cancer in patient-derived xenograft mice Inhibition of Wnt target gene expression disrupts cancer stem cell function	[198]

#### Anti-CD44 mAb

CD44 is a widely recognized colorectal CSC marker, and inhibition of CD44 can suppress the growth and invasion of CSCs and sensitize tumors to therapy [190]. However, in a phase 1 clinical trial for patients with advanced solid tumors, RG7356 (a humanized anti-pan-CD44 mAb) was terminated early because it did not demonstrate a clinical, pharmacodynamic dose‒response relationship, although RG7356 demonstrated acceptable safety. Among the 65 oncology patients participating in the clinical trial, 19 were CRC patients. The final results showed that most patients showed disease progression after the second cycle, with only 13 of 61 patients that could be evaluated (21%) showing the best response, which was stable disease (SD), and with tumor shrinkage observed in 3 of the CRC patients [191].

#### Anti-Lgr5/RSPO mAb

Lgr5 is a colorectal CSC marker associated with the Wnt/β-catenin pathway, and anti-Lgr5 mAbs that recognize colorectal CSCs have been developed [192]. Furthermore, BNC101, a mAb with high affinity for Lgr5, exhibited antitumor activity that led to prolonged survival in multiple mouse models with CRC patient-derived xenograft (PDX) tumors [193, 194]. In animal experiments, BNC101 has been shown to increase the efficacy of anti-PD-1 therapy [195]. R-spondin (RSPO) is a ligand of Lgr5, and their combination significantly enhanced the activity of the Wnt/β-catenin signaling pathway [196]. Targeting RSPO with antibodies inhibited the growth of various tumors, including CRC, and inhibited tumor stemness [197]. Storm et al. used a synthetic anti-RSPO3 mAb to treat PTPRK-RSPO3 fusion-positive CRC cells in PDX mouse models, which effectively inhibited tumor growth and promoted tumor differentiation. An RNA sequencing data analysis showed that the expression of Wnt target genes was obviously reduced after this mAb treatment. Moreover, the number and function of CSCs were also reduced [198]. DBPR117, an anti-RSPO3 antibody, is being studied in combination with ICIs in solid tumors[199].

#### Anti-Notch/DLL mAb

The Notch signaling pathway is important to colorectal CSC self-renewal and resistance to apoptosis [200]. Accordingly, it is a direction to design mAbs targeting Notch receptors or DLL ligands for blocking Notch downstream genes.

Notch receptors, including Notch 1, Notch 2, Notch 3 and Notch 4, are expressed in human cells. Knockdown of Notch1 in CRC cells inhibited their growth and proliferation [201]. The effectiveness of brontictuzumab, a mAb designed to target Notch1 [202], has been evaluated in a phase 1 clinical trial in patients with solid tumors. Forty-eight patients, including 14 patients with CRC, were enrolled in this study. Although brontictuzumab was well tolerated, only 6 of the 36 evaluable patients showed clinical benefit [203]. Tarextumab is a cross-reactive mAb developed to block Notch2 and Notch3 simultaneously. In PDX models of breast cancer, lung cancer, ovarian cancer or pancreatic cancer, the combination of tarextumab and chemotherapeutic drugs showed positive antitumor activity, and the number and tumorigenic capacity of the CSCs in residual tumors were significantly decreased after treatment [204]. However, in a phase I clinical trial of solid tumors using tarextumab alone, the final therapeutic effect of the 42 patients enrolled, 9 of whom were CRC patients, was not obvious, although the safety of the drug was verified [205]. In summary, evaluation of the therapeutic effect of the tarextumab–chemotherapy combination was promising in clinical trials.

Among the six ligands that bind Notch, DLL4 is vascular specific and involved mainly in the physiological process of angiogenesis and arteriogenesis [206]. An anti-DLL4 mAb in colorectal tumor-bearing mice inhibited tumor cell proliferation and reduced tumor volume [207]. In another study, an anti-DLL4 mAb was shown to be effective against both *KRAS*-wild-type and *KRAS*-mutant CRC cell lines. In addition, the combination therapy of an anti-DLL4 mAb with irinotecan effectively reduced the number of CSCs in CRC tissues [208].

#### Other mAbs

Xu et al. developed a mAb (CC188) that recognized a carbohydrate epitope on the cell surface of colorectal CSCs. CC188 showed adequate sensitivity and specificity for human CRC cells in a tissue microarray analysis. In addition, CC188 inhibited the invasion of human CRC cells [209].

CRC cells secrete progastrin in an autocrine manner, which promotes colorectal CSC self-renewal and maintenance. Inhibiting the expression of progastrin markedly reduced the number and impaired the tumorigenic capacity of colorectal CSCs [210]. The results of in vivo and in vitro experiments showed that Hz8CV2, an mAb targeting progastrin, inhibited the survival and self-renewal of colorectal CSCs and blocked tumor growth, invasion and metastasis. A mechanistic investigation revealed that Hz8CV2 inhibited the Wnt/β-catenin signaling pathway. Furthermore, the combination of Hz8CV2 and 5-Fu showed encouraging therapeutic effects compared to those of 5-Fu alone, confirming Hz8CV2 as a potential new treatment for colorectal tumors and CSCs [211].

### Antibody‒drug conjugates (ADCs)

Although mAbs have provided great benefits to cancer patients in the past two decades, the use of mAbs still has limitations, including the drug resistance acquisition and ineffective treatment [212]. In addition, the ability of mAbs alone to kill tumor cells is not satisfactory [213]. Therefore, ADCs are designed as new synthetic drug molecules that which consist of three parts: a monoclonal antibody, chemical linker and cytotoxic payload [214, 215]. The antibody is responsible mainly for recognizing specific antigens on the tumor cell surface and being internalized into tumor cells where the payload is delivered [216]. The chemical linker is the bridge connecting the mAb to the cytotoxic payload and is responsible for the release of the cytotoxic payload [217]. The cytotoxic payload kills tumor cells after entry of the ADC into tumor cells [218]. To date, more than one dozen ADC drugs have been approved for marketing worldwide, and more than one hundred ADC drug candidates are in the clinical evaluation stage [219]. In terms of ADCs that target and kill colorectal CSCs, a variety of drugs are being developed and entered into clinical trials **(**Table 2**)**.

**Table 2:** Elimination of colorectal CSCs based on antibody–drug conjugates

mAb	Cytotoxic payload	Clinical trial title	References
Anti-CD166	DM4	PROCLAIM-CX-2009: A Trial to Find Safe and Active Doses of an Investigational Drug CX-2009 for Patients With Selected Solid Tumors (ID: NCT03149549)	[228]

mAb	Cytotoxic payload	Action	References
Anti-Lgr5	MMAE	In vivo experiments significantly reduce tumor volume and eliminate colorectal CSCs with less intestinal toxicity and prolonged survival of tumor mice	[220, 221]
Anti-Lgr5	NMS818	Exhibits target-dependent toxicity while having antitumor activity	[221]
Anti-CD133	SN-38	Targeted killing of colorectal CSCs, delaying tumor recurrence	[222]
Anti-CD44	Doxorubicin	With stronger affinity to tumor tissue, C26 tumor-bearing mice have a longer survival period after treatment	[224]
Anti-EpCAM (3–17I)	Saporin	Stronger lethality to EpCAM-positive colorectal cancer cells Becomes more effective with increasing light dose	[225]
Anti-EpCAM (HEA125)	α-amanitin	In vivo experiments inhibit tumor growth and reduce side effects	[226]

#### Lgr5

As a recognized marker of colorectal CSCs, Lgr5 is an important target for the elimination of CSCs by ADCs. Gong et al. developed two ADCs targeting Lgr5 to eliminate CSCs. This team utilized cleavable and non-cleavable chemical linkers to conjugate the anti-Lgr5 mAb with the tubulin-inhibiting agent monomethyl auristatin E (MMAE), in which yielded anti-LGR5-mc-vc-PAB-MMAE and anti-LGR5-mp-MMAE, respectively. Subsequent in vitro experiments revealed that anti-LGR5-mc-vc-PAB-MMAE with a cleavable chemical linker was more cytotoxic than anti-LGR5-mp-MMAE. In vivo experiments also proved that anti-LGR5-mc-vc-PAB-MMAE significantly reduced tumor volume, and it eliminated CSCs, achieving complete remission (no tumor detected), without inducing toxicity to the normal portion of the intestinal tract [220]. Another team designed two other ADC drugs targeting Lgr5, namely, anti-LGR5–mc-vc-PAB–MMAE and anti-LGR5–NMS818. MMAE is an antimitotic drug, while NMS818 contains the components of the topoisomerase inhibitor anthracycline drug PNU159682, which is a DNA-damaging drug. Both ADC drugs showed significant antitumor activity against CRC and CSCs and prolonged survival in animal models. Anti-LGR5–mc-vc-PAB–MMAE showed better tolerance, while anti-LGR5–NMS818 showed target-dependent toxicity, which may be related to the bystander effect. A possible mechanism underlying the difference in toxicity involves NMS818 released in tumor cells penetrating the cell membrane to affect surrounding normal cells, whereas free MMAE exerted a 10–100-fold weaker effect than NMS818 on dividing cells [221]. Overall, these investigations targeting Lgr5 via ADCs indicate viable therapeutic strategies to obliterate colorectal CSCs.

####  CD133

Ning et al. developed CD133Ab-NPs-SN-38 nanoparticles, which combine the anti-CD133 mAb and SN-38, an inhibitor of topoisomerase I. Compared with free SN-38 and nanoparticles loaded with SN-38 (NPs-SN-38), CD133Ab-NPs-SN-38 showed greater antitumor ability and precisely targeted and killed colorectal CSCs, inhibited tumor growth and delayed tumor recurrence, demonstrating that CD133 is a promising ADC target for colorectal CSC elimination [222].

#### CD44

As the first nanodrug approved by the FDA, Doxil is loaded with doxorubicin encapsulated in liposomes, and it shows a longer circulation time and induces fewer side effects [223]. Arabi et al. used an anti-CD44 mAb to modify Doxil and thus produced CD44-Doxil for the targeted elimination of colorectal CSCs. Because of the engagement of the anti-CD44 mAb, CD44–Doxil showed higher affinity for tumor tissue, with a higher T/NT ratio than Doxil alone. Both in vitro and in vivo experiments showed significant antitumor effects, and C26 tumor-bearing mice treated with CD44-Doxil presented with longer survival times [224].

####  EpCAM

EpCAM is a marker of colorectal CSCs, and a biotinylated anti-EpCAM mAb (3–17I) was linked to saporin (a ribosome-inactivating toxin) to form 3–17I-saporin. 3-17I-Saporin showed an enhanced killing effect on to the EpCAM-positive WiDr CRC cell line. Due to the involvement of photochemical internalization (PCI), 3–17I-saporin became more potent with increasing doses of light stimulation [225]. Exploiting another anti-EpCAM mAb, HEA125, and toxic α-amanitin, a specific inhibitor of RNA polymerase II that is closely related to CRC, Liu et al. designed ama–HEA125 via conjugation of the HEA125 mAb to α-amanitin. Ama–HEA125 effectively inhibited tumor growth in CRC-bearing mice with a low α-amanitin dosage and thus showed greatly reduced side effects [226].

#### CD166

CD166 has also been identified as a marker of colorectal CSCs [227]. CX-2009 is an ADC drug that combines an anti-CD166 mAb Probody with the maytansinoid derivative DM4. A phase I/II clinical trial was conducted to evaluate the safety, pharmacokinetics and efficacy of CX-2009 for use in patients with advanced solid tumors [228]. In another preclinical study, CX-2009 was combined with anti-PD-1 and used to treat CD166-positive CT26 cell-bearing mice, and the results showed that the combined treatment significantly inhibited the growth of the tumors in the mice. In addition, CX-2009 reduced the number of exhausted CD8^+^ T cells and promoted the activation of T cells, possibly because of the involvement of anti-PD-1 [229].

### Bispecific antibodies

To prevent the ubiquitous immune evasion of tumor cells and CSCs [230], new therapeutic strategies have been developed to combine immune cells with tumor cells [231]. Bispecific antibodies (BsAbs) are artificially constructed hybrid proteins and are considered potential antitumor drugs [232]. In contrast to traditional mAbs that can recognize only a single epitope, BsAbs were capable of simultaneously recognizing two different antigen epitopes [233]. Bispecific T-cell engager (BiTE) is a type of BsAb that links T cells and tumor cells together to enhance their antitumor effects [234]. Currently, more than 50 BsAbs are undergoing clinical trials [235], and there are also attempts to target colorectal CSCs **(**Table 3**)**.

**Table 3:** Elimination of colorectal CSCs based on bispecific antibodies

Targets	Drugs	Clinical trial title	References
EpCAM × CD3	MT110	Phase I Study of MT110 in Lung Cancer (Adenocarcinoma and Small Cell), Gastric Cancer or Adenocarcinoma of the Gastro-Esophageal Junction, Colorectal Cancer, Breast Cancer, Hormone-Refractory Prostate Cancer, and Ovarian Cancer (MT110-101) (ID: NCT00635596)	[237]
EpCAM × CD3	Catumaxomab	Study in EpCAM Positive Patients With Symptomatic Malignant Ascites Using Removab Versus an Untreated Control Group (ID: NCT00836654)	[241]
DLL4 × VEGF	Dilpacimab (ABT-165)	A Study of ABT-165 Plus FOLFIRI vs Bevacizumab Plus FOLFIRI in Subjects With Metastatic Colorectal Cancer Previously Treated With Fluoropyrimidine, Oxaliplatin and Bevacizumab (ID: NCT03368859)	[244]
DLL4 × VEGF	CTX-009 (ABL001)	This is a Study to Evaluate the Safety and Tolerability of the Study Drug ABL001, and to Determine the Maximum Tolerated Dose and/or Recommended Phase 2 Study Dose of ABL001 (ID: NCT03292783)	[246]
DLL4 × VEGF	CTX-009 (ABL001)	A Study of CTX-009 in Adult Patients With Metastatic Colorectal Cancer (ID: NCT05513742)	[247]
EGFR × LGR5	MCLA-158	Inhibiting the growth of CRC organoids Efficacy in both *Ras* wild-type and *Ras* mutant PDX mice	[250]

Targets	Drugs	Action	References
EGFR × LGR5	MCLA-158	Inhibiting the growth of CRC organoids Efficacy in both *Ras* wild-type and *Ras* mutant PDX mice	[250]

####  EpCAM × CD3 BiTE

MT110 and MuS110 are BiTEs linking anti-EpCAM and anti-CD3 antigen-specific antibody single chains together, with MT110 is human-derived and MuS110 is mouse-derived. MT110 exhibits high antitumor activity against the human CRC cell line SW480 in tumor-bearing mice, with a vigorous ability to inhibit tumor initiation, demonstrating the CSC-targeting capacity of MT110. MuS110 showed the same therapeutic effect in a mouse lung metastasis model of CT26 cells [236]. In a phase I clinical trial of MT110 (solitomab) in the treatment of refractory solid tumors, dose-limiting toxicities occurred in 15 of 65 patients enrolled. Among 54 patients who were assessed by RECIST, 17 patients (31%) had the best SD response [237]. In another study, the combination of bispecific EpCAM × CD3 antibody and umbilical cord blood mononuclear cells (MNCs) derived from mouse liver inhibited tumor growth in SW480 tumor-bearing mice [238]. Catumaxomab is the first commercially marketed trifunctional antibody (anti-EpCAM × anti-CD3). In addition to binding tumor cells and T cells, its Fc region can also connect to helper cells by binding to Fcγ receptors [239]. In clinical trials of patients with malignant ascites, including CRC, catumaxomab showed an acceptable safety and promising efficacy [240, 241]. Interestingly, intraperitoneal injection of catumaxomab facilitates the accumulation of CD8^+^ T cells in the peritoneal cavity [242].

#### DLL4 × VEGF

Dilpacimab (ABT-165) is a BsAb targeting DLL4 and VEGF. In preclinical studies, the therapeutic effect of Dilpacimab on tumor-bearing mice of human CRC SW-48 cells was superior to that of anti-DDL4 or anti-VEGF mAb alone. In addition, dilpacimab combined with chemotherapy had a better curative effect on tumor-bearing mice with the human CRC cell line HT-29 than chemotherapy alone [243]. However, in a phase II clinical trial, the combined efficacy of dilpacimab and FOLFIRI chemotherapy in patients with metastatic CRC was investigated. The results showed that dilpacimab combined with FOLFIRI chemotherapy did not bring significant clinical benefit, and the clinical trial was terminated early [244].

As another BsAb targeting DLL4 and VEGF, CTX-009 (ABL001) showed antitumor activity in xenograft mouse models of human CRC cells Colo205, WiDr, SW-48 and SW620. The combined treatment of CTX-009 and irinotecan on SW48 and SW620 xenograft mice showed a synergistic effect, and their combination reduced the expression of DLL4 in tumor tissue and promoted the apoptosis of tumor cells [245]. In a phase Ia clinical trial, patients with metastatic gastrointestinal tumors were treated with CTX-009, and the safety and preliminary efficacy of CTX-009 were verified [246]. Another phase II clinical trial of CTX-009 is ongoing in adult patients with metastatic CRC who have received at least two types of systemic chemotherapy [247].

#### EGFR × LGR5

Cetuximab, a mAb targeting EGFR, is the first-line drug for the treatment of patients with *Ras* wild-type CRC. However, cetuximab still has therapeutic limitations, and it is difficult to produce curative effect on patients with *Ras* mutant CRC [248]. MCLA-158, a BsAb targeting EGFR and Lgr5, showed significant inhibitory effects on patient-derived CRC organoids in a preclinical study [249]. Compared with cetuximab alone, MCLA-158 showed better efficacy on both *Ras* wild-type and *Ras* mutant CRC in PDX mouse models, and MCLA-158 can effectively target CSCs and inhibit the initiation of tumor organoids [250].

### Tumor vaccine

With the rise of tumor immunotherapy represented by ICIs and CAR-T cells, the research on tumor vaccines has ushered in a revival [251]. Tumor vaccines carrying tumor-associated antigens (TAAs) or tumor-specific antigens (TSAs) are recognized by the human immune system, and antitumor immune responses, mainly T-cell-based cellular immunity, are triggered to prevent the propagation of tumors [252]. As early as 1985, clinical research on the treatment of CRC with tumor vaccines was reported [253]. Tumor vaccines based on CSCs have been proven to have significant antitumor ability [254]. For CRC, attempts to achieve immune prevention and treatment of CSCs through tumor vaccines are underway, which may break through the plight of the suppressive immune microenvironment of CRC.

#### MUC1 vaccine

Studies have shown that high expression of MUC1 is associated with poor prognosis in CRC patients [255]. MUC1 is significantly enriched in colorectal CSCs and is also considered a potential target for intervention in colorectal CSCs [256]. A study showed that MUC1 is a key component of a CSC vaccine that exerts antitumor immune activity. When comparing the tumor prevention effect of vaccines generated from control CSC lysate and shMUC1 CSC lysate, the vaccine developed from CSC lysate showed a significant preventive and protective effect on SW620 cells, while knocking down MUC1 impaired the protective effect [257]. In contrast, another study designed a CSC vaccine with high expression of MUC1. Mice injected with this vaccine showed more robust antitumor ability after receiving CT26 cell shock, characterized by decreased CSCs in tumor tissue, enhanced infiltration and cytotoxicity of NK, CD4^+^ and CD8^+^ T cells, increased antibody production, and reduced immunosuppressive MDSC and Treg cells [258].

Tecemotide is a liposomal vaccine based on the 25 core amino acids of MUC1 [259]. In a clinical trial of tecemotide in the treatment of CRC patients with liver metastases after resection, the median OS of the patients was up to 62.8 months [260]. Compared with the other two clinical trials of cetuximab and bevacizumab combined with chemotherapy, tecemotide vaccinated patients had a longer survival period [261].

#### DC vaccine

As the most powerful antigen-presenting cells in the body, DCs are specifically responsible for antigen presentation to T cells to activate antitumor immunity. Therefore, tumor antigen (TAA or TSA)-loaded DCs serve as a rational vaccine for tumor targeting [262]. Fu et al. loaded colorectal CSC lysate onto DCs to make a DC vaccine, which had a significant inhibitory effect on tumor growth in colorectal CSC-bearing mice models. In addition, mice vaccinated with DC vaccine in advance produced more interferon after receiving CSC shock to inhibit the occurrence of tumors [263]. In another study, colorectal CSC-derived defective ribosomal products containing autophagosome-rich blebs (DRibbles) were cocultured with DCs to make DRibble vaccines, and treatment of BALB/c CRC mice with the DRibble vaccine resulted in more CD8^+^ T-cell-mediated cytotoxicity and longer survival [264].

### Adoptive cell therapy

Adoptive cell therapy transforms and activates autologous or allogeneic immune cells in vitro and then expands and infuses them back into the patient's body to eliminate tumor cells [265]. A study carried out as early as 1988 showed that immune cells extracted from melanoma patients could mediate the objective regression of melanoma in some patients after expansion in vitro, combination with IL-2 and infusion back into the patient [266]. At present, cell adoptive therapy has been developed into various methods such as CTL [267], CAR-T [268], TCR-T [269], and CAR-NK [270]. Overall, the number and status of immune cells affect the prognosis and survival of CRC patients with adoptive cell therapy [271, 272]. Targeting and eliminating colorectal CSCs through adoptive cell therapy is emerging as a direction for exploring personalized treatment of CRC **(**Table 4**)**.

**Table 4:** Elimination of colorectal CSCs based on adoptive cell therapy

Type	Target	Action	References
CTL	OR7C1 ASB4 CEP55	Effective recognition and killing of colorectal CSCs	[273] [274] [275]
CAR-T	EpCAM CD133 DCLK1 Lgr5	Recognition and killing of colorectal CSCs without antigen presentation by MHC-I molecules	[277, 278] [279] [50, 280] [281]
CAR-NK	EpCAM	EpCAM-specific CAR-NK can effectively recognize and kill colorectal CSCs Combined with regorafenib to exert a stronger anti-tumor effect	[286]

#### CTL

A study showed that OR7C1 is a potential marker of colorectal CSCs, and its high expression enhanced the stemness of CRC. The induction and expansion of OR7C1-specific CTLs in vitro proved to be effective in recognizing and eliminating colorectal CSCs, and these results were confirmed by in vivo experiments [273]. Another study revealed that the specific CTL induced by the peptide epitope of ASB4, which is expressed in tumor tissues but not in normal tissues, can distinguish colorectal CSCs from normal stem cells, leading to colorectal CSC-specific lethality. Moreover, CSC-specific CTLs also inhibited tumor growth in vivo, demonstrating the clinical efficacy of targeting and eliminating colorectal CSCs [274]. In addition, CEP55-specific CTLs have also been shown to be able to target and kill colorectal CSCs, which may present another strategy for CRC treatment after chemotherapy resistance [275].

#### CAR-T

CAR-T cells are genetically modified T cells expressing CARs, which can recognize and kill tumor cells without the contribution of MHC-I molecule antigen presentation [276]. Anti-EpCAM CAR-T cells targeting epithelial cell adhesion molecule (EpCAM) inhibited the progression of CRC in tumor-bearing mice without inducing obvious toxic side effects [277, 278]. Anti-CD133 CAR-T cells showed strong antitumor activity against CD133^+^ colorectal CSCs, and their safety and preliminary efficacy have been verified in a phase I clinical trial [279]. Anti-DCLK1 CAR-T cells, which targeted the colorectal CSC marker DCLK1, effectively recognized CSCs and released IFN-γ, which exerted cytotoxic functions. In vivo experiments showed that anti-DCLK1 CAR-T-cell treatment of LOVO xenograft mice inhibited tumor growth by more than 42% without inducing obvious toxicity [280]. The efficacy of CNA3103, CAR-T cells targeting Lgr5 antigen, were evaluated in a 1/2a clinical trial for patients with metastatic CRC [281]. In addition, the combination of anti-PD-L1 CAR-T and DC vaccines achieved good preclinical efficacy in targeting ALDH1^+^ colorectal CSCs, reflecting the value of combined immunotherapies [282].

#### CAR-NK

NK cells are another type of immune cells which can effectively recognize and kill tumor cells. The combination of CAR and NK cells is a novel attempt at generating antitumor immunotherapy with superior safety [283]. NK cells have been shown to preferentially target CSC populations [284, 285]. The combination therapy consisting of EpCAM-specific CAR-NK cells and regorafenib showed stronger antitumor activity than monotherapy against CRC cells and tumor-bearing mice. EpCAM-specific CAR-NK cells effectively recognized EpCAM^+^ CRC cells and released IFN-γ, perforin and granzyme B to induce cytotoxicity [286].

### Small molecule inhibitors

Small molecule inhibitors have become the main drugs approved by FDA for anti-tumor therapy due to their advantages of high selectivity, convenience, wide efficacy, and high tissue permeability [287]. Small molecule inhibitors are also one of the important targeting strategies for colorectal CSCs, mainly including targeting CSC surface markers and stemness pathways.

DCLK1-IN-1 is a highly selective DCLK1 kinase inhibitor that has been shown in multiple experiments to inhibit the aggressiveness and stemness of colorectal cancer and reverse chemotherapy resistance [50, 51, 288]. In addition, high expression of DCLK1 is associated with low CD8^+^ T cell infiltration [166], and one study has found that the use of DCLK1-IN-1 in renal cell carcinoma can promote the efficacy of ICIs [289], which provides new ideas for the treatment of CRC. Napabucasin is a CSC inhibitor that targets STAT3. It has been tested in Phase III clinical trials in patients with metastatic CRC. Although the results showed no significant difference in OS between the Napabucasin group and the placebo group, targeting pSTAT3 positive patients, the Napabucasin group had better survival [290]. Jing B et al. used apoptotic tumor-derived particles (TMPs) as the carrier to construct nanodrugs loaded with Napabucasin N3-TMPs@NAP, which showed strong anti-tumor immune activity in vivo [291]. Mithramycin A (Mit-A) is another small molecule inhibitor that has been shown to inhibit colorectal CSCs. Dutta R et al. found that Mit-A can increase the expression of PD-L1 in CRC tissues, thereby increasing the drug sensitivity of anti-PD-L1 therapy. Combined treatment with Mit-A and anti-PD-L1 can increase the infiltration of CD8 + T cells and reduce the immunosuppressive Treg cells [292].

## Perspectives and conclusions

At present, there are some challenges in CSC targeting, including specificity, heterogeneity and plasticity. First, many markers and targets of CSCs are TAAs but not TSAs; therefore, immunotherapy against these targets leads to side effects. Second, there are many subgroups of CSCs, and different subgroups have specific gene expression patterns, making the identification of universal targets in all subgroups difficult. Third, after elimination of CSCs, non-CSCs can dedifferentiate into CSCs because of the plasticity of these cells, and this process is similar to process underlying relapse in patients who had received traditional therapy; in summary, after elimination of non-CSCs, the surviving CSCs differentiate into new non-CSCs to generate new tumors [293]. Hence, it may be feasible to overcome the plasticity of CSCs and target their microenvironment, which CSCs need to survive. With the development of new biotechnologies, such as RNA sequencing, single-cell sequencing and CRISPR screening [294], our understanding of CSCs will be further advanced, and new promising targets for immunotherapy targeting of CSCs will certainly be identified.

Although tumor immunotherapy has achieved great success in a variety of tumors, and emerges as a rising star in tumor targeting even for solid tumors, there are still many dilemmas for immunotherapy, including cytokine storm, impaired tumor-infiltration of immune cells, immunosuppressive microenvironment, immune tolerance and relapse [295–298]. CSCs play important roles in the remodeling of the immunosuppressive microenvironment and tumor cell immune escape, which largely account for tumor relapse and drug resistance after immunotherapy. However, the current investigation on tumor microenvironment is often population-based, and the specific microenvironment of CSCs has been less extensively studied. Due to the key role of CSCs in tumor initiation, metastasis, drug resistance and relapse, there is an urgent need to understand the specific CSC microenvironment. Some recently advanced technologies, such as genetic labeling of CSCs, microenvironment labeling, CSC and immune cell coculturing systems, CRISPR screening technology and spatial transcriptome assays [299], will greatly promote investigation of the interaction between CSCs and immune cells, ultimately providing a theoretical basis for CSC immune targeting.

Traditionally, CRC has been considered a “cold” tumor with insufficient infiltration of immune cells and therefore not suitable for classical ICI or CAR-T therapy. However, in some CRC patients, especially patients with high microsatellite instability (MSI), tumor immunotherapy has been effective, indicating the importance of the immune microenvironment in immunotherapy. Therefore, how to remodel the tumor immune microenvironment from “cold” to “hot” is the key to tumor immunotherapy, and definitely it will provide new insight for tumor immunotherapy to intensively investigate the mutual interaction between CSCs and immune cells. Moreover, the success of tumor immunotherapy, similar to that traditional therapy, is reduced by many difficulties, such as limited drug delivery, loss of targeting ability, drug tolerance and disease relapse. A variety of interdisciplinary strategies have been established for the delivery of traditional drugs to promote their stability and tumor-specificity, as well as to reduce side effects [300]. Similarly, interdisciplinary strategies will bring breakthrough to tumor immunotherapy, which needs to be further strengthened in future. In summary, immunotherapy of patients with CRC and colorectal CSCs requires the integration of strategies used in multiple disciplines, including biology, medicine, biomaterials and nanotechnology.

## Data Availability

Not applicable.

## Abbreviations

CRC: Colorectal cancer
ISC: Intestinal stem cell
APC: Adenomatous polyposis coli
Axin: Axis ihibition
GSK3β: Glycogen synthase kinase-3 beta
CK1α: Casein kinase 1α
DVL: Dishevelled
TCF: T-cell factor
LEF: Lymphatic enhancer factor
EMT: Epithelial-mesenchymal transition
JAK: Janus kinase
STAT: Signal transducers and activators of transcription
DLL: Delta-like ligand
NICD: Notch intracellular domain
STRAP: Serine-threonine kinase receptor-associated protein
ncRNA: Non-coding RNA
Dnmt: DNA methyltransferases
siRNA: Short interfering RNA
NuRF: Nucleosome remodeling factor
NuRD: Nucleosome remodeling and deacetylase
miRNA: MicroRNA
lncRNA: Long noncoding RNA
circRNA: Circular RNA
ICI: Immune checkpoint inhibitor
TCR: T-cell receptor
NKG2D: Natural killer cell group 2D
ITIM: Immune receptor tyrosine–based inhibitory motif
ITSM: Immune receptor tyrosine–based switch motif
PBMC: Peripheral blood mononuclear cell
TME: Tumor microenvironment
TAMM: Tumor-associated monocyte and macrophage
TADC: Tumor-associated dendritic cell
MDSC: Myeloid-derived suppressor cell
DCLK: Doublecortin-like kinase
mAb: Monoclonal antibody
SD: Stable disease
CAR: Chimeric antigen receptor
PDX: Patient-derived xenograft
ADC: Antibody‒drug conjugate
MMAE: Monomethyl auristatin E
PCI: Photochemical internalization
BsAb: Bispecific antibody
TAA: Tumor-associated antigen
TSA: Tumor-specific antigen
